# Monitoring the Age of Mosquito Populations Using Near-Infrared Spectroscopy

**DOI:** 10.1038/s41598-018-22712-z

**Published:** 2018-03-27

**Authors:** Ben Lambert, Maggy T. Sikulu-Lord, Vale S. Mayagaya, Greg Devine, Floyd Dowell, Thomas S. Churcher

**Affiliations:** 1 Department of Zoology, University of Oxford, South Parks Road, Oxford, OX1 3PS UK; 2 MRC Centre for Outbreak Analysis and Modelling, Infectious Disease Epidemiology, Imperial College London, London, W2 1PG UK; 3 Queensland Alliance of Agriculture and Food Innovation, The University of Queensland, Brisbane, Queensland Australia; 4 Ifakara Health Institute, Biomedical Unit, Ifakara and Dar es Salaam Branches, Ifakara and Dar es Salaam, Tanzania; 5 Mosquito Control Laboratory, QIMR Berghofer Medical Research Institute, Brisbane, Queensland Australia; 6 USDA, Agricultural Research Service, Center for Grain and Animal Health Research, 1515 College Avenue, Manhattan, KS 66502 USA

## Abstract

Mosquito control with bednets, residual sprays or fumigation remains the most effective tool for preventing vector-borne diseases such as malaria, dengue and Zika, though there are no widely used entomological methods for directly assessing its efficacy. Mosquito age is the most informative metric for evaluating interventions that kill adult mosquitoes but there is no simple or reliable way of measuring it in the field. Near-Infrared Spectroscopy (NIRS) has been shown to be a promising, high-throughput method that can estimate the age of mosquitoes. Currently the ability of NIRS to measure mosquito age is biased, and has relatively high individual mosquito measurement error, though its capacity to rigorously monitor mosquito populations in the field has never been assessed. In this study, we use machine learning methods from the chemometric literature to generate more accurate, unbiased estimates of individual mosquito age. These unbiased estimates produce precise population-level measurements, which are relatively insensitive to further increases in NIRS accuracy when feasible numbers of mosquitoes are sampled. The utility of NIRS to directly measure the impact of pyrethroid resistance on mosquito control is illustrated, showing how the technology has potential as a highly valuable tool for directly assessing the efficacy of mosquito control interventions.

## Introduction

Mosquito borne diseases remain a major cause of suffering and death. Malaria is thought to have killed 429,000 people in 2015^1^, whilst in 2013 it was estimated that there were 58·4 million symptomatic dengue virus infections^2^. Killing adult mosquitoes is, therefore, a key public health intervention, being the primary method of control for malaria, dengue, chikungunya and Zika. Despite this, there are no simple entomological methods that can directly evaluate the efficacy of mosquito control programmes. Mosquito abundance fluctuates substantially from day to day and according to location, making population size estimates imprecise. More importantly, the number of mosquitoes in itself is a poor predictor of disease transmission. For diseases such as dengue, there is no established relationship between *Aedes aegypti* abundance and transmission, and the size of *Anopheles* populations can appear stable, despite the implementation of effective malaria control programs. The age structure of a vector population is theoretically a far more powerful predictor of transmission. Most mosquito borne pathogens and parasites have a long extrinsic incubation period (EIP, the time between when a mosquito is infected and becomes infectious). For the malaria parasites, it is typically >10 days^3^, whilst for the dengue and Zika viruses it is >7 days^4,5^. Given that many adult mosquito vectors live for an average of <10 days^6^, then even in the absence of vector control, it is only the rarer, older female mosquitoes that transmit the infection. Simple measures of abundance are, therefore, insufficient to determine transmission risks or characterise the impact of vector control programmes. In the past, the potential impacts of vector control have been related to the coverage of the human population, such as the numbers sleeping under treated bednets or living in houses protected by larviciding, fumigation or residual insecticides. However, resistance to the most commonly-used insecticides for control of *Ae*. *aegypti* and many Anophelines is now widespread, hence breaking the link between coverage and protection; rendering intervention coverage a poor predictor of efficacy.

It has long been recognised that mosquito age is potentially a good predictor of the efficacy of vector control, though there are no simple reliable methods for its measurement in the field. The current best method for estimating mosquito survival (and hence indirectly estimating mean age) in the wild is mark-release-recapture studies. By releasing a known number of marked mosquitoes and monitoring the number recaptured over time, estimates can be made of the combined mortality and dispersion rate. However, these experiments are time-consuming and it can be costly to release the large numbers of marked mosquitoes required to achieve a reasonable level of estimation accuracy^7^. Release and recapture studies also incorporate their own bias through their manipulation of mosquito behaviour and their unintended impacts on survival. A more direct way of estimating mosquito age is to dissect the ovaries and assess whether (and how many times) a female has laid eggs^8^. This method is very laborious, rendering it impractical to use on a programmatic scale and doesn’t directly indicate biological age (just the number of feeding cycles, the length of which might vary with vector control). In some species, for example, *Ae*. *aegypti*, it is not easy to estimate age from the number of gonotrophic cycles a mosquito has undergone because of multiple feeding events, continual egg production and reabsorption. A number of alternative techniques have been developed to age mosquitoes, including the analysis of changes in cuticular hydrocarbons^9^, gene transcription^10^, proteins^11^, pteridine fluorescence and near-infrared spectroscopy (NIRS)^12^. Unlike the others, NIRS is a rapid, non-destructive method, simple to use and does not consume reagents. Large numbers of mosquitoes can be processed relatively economically, making it feasible to use NIRS to routinely assess the efficacy of vector control interventions.

NIRS works by measuring the change in absorbance of light at different wavelengths by organic compounds within the mosquito head and thorax. Mosquitoes of known ages are scanned and a simple machine learning algorithm is then used to convert spectral data into estimates of biological age, which can then be validated against blinded samples. Like all the above methods, NIRS has considerable measurement error, and predictions of individual mosquito age are therefore relatively poor. Researchers typically use NIRS to define mosquitoes as young (<7 days) or old (≥7 days)^12–16^. With this simplification, NIRS could predict the percentage of young or old mosquitoes with 78% to 90% accuracy^12–16^. From a practical perspective, NIRS predictions appear to have no significant loss of precision for mosquitoes killed earlier and kept in RNA*later* or other preservation methods^14,17^. Equally, in the laboratory, mosquito age was not noticeably impacted by the physiological status of the mosquito when head and abdomens were scanned^18^ (i.e. whether it had mated, blood fed and laid eggs), or whether it had been exposed to pyrethroid insecticide^16^.

To date, all mosquito aging techniques which estimate age on a continuous scale have been evaluated on their ability to predict individual mosquito age. When monitoring vector control intervention in the field, the overall mean and age distribution across the mosquito population is more important than the age of any individual mosquito. By simply sampling more mosquitoes, even relatively imprecise metrics on the individual-level can generate highly accurate population-level estimates. These may be sufficiently precise to enable NIRS to be used to evaluate and prioritise mosquito control interventions.

**Figure 1:**
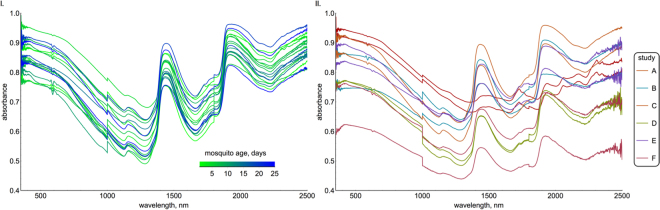
Illustration how mosquito absorbance varies with age within a single study (**I**) and between studies (**II**). I. Spectra from 34 mosquitoes scanned in the same study (Study A in Table 1) showing the intra-study variation, with the line colour representing the true age of the mosquito (green = young, blue = old). II. How near infrared absorbance of 7 day old mosquitoes varies with wavelength, indicating inter-study variability, with each line coloured according to the study the spectra was drawn from.

Relatively simple machine learning algorithms (GRAMS IQ^19^) have been used to convert spectral data into predictions of mosquito age (here referred to as the ‘standard NIRS chemometric method’). Here we collate data from several published studies that assessed the ability of NIRS to age *Anopheles gambiae sensu lato* (*Anopheles gambiae* s.s. and *Anopheles arabiensis*) and *Ae*. *aegypti* mosquitoes, and used it to determine whether advances in machine learning methodology (referred to as the ‘iPLS NIRS chemometric method’) can improve the precision of individual mosquito age estimates. An illustration of how mosquito absorbance varies with age is given in Fig. 1, whilst Table 1 summarises the datasets investigated (see Table 1 caption and Methods for experimental details). Simulation is then used to measure how the accuracy of the improved machine learning approach for estimating population mean age depends on the number of mosquitoes in the training dataset (mosquitoes with known ages used to calibrate the model), and the number in the test dataset (mosquitoes randomly sampled from the wild with unknown age). The utility of the method is illustrated using a theoretical example, to show how NIRS could be used to investigate whether pyrethroid resistant mosquitoes are impeding malaria control.

**Table 1:** A summary of the mosquito age data showing the different mosquito species, country of origin, sex, and preservation methods. The raw NIRS data is available in the supplementary materials to this paper. The data for experiment B are from^14^; the data for experiments C, D, F, G, H, I is from^12^; the data for experiments A and E are not yet published although the experimental protocol is the same as presented in^12^; the data for J are from^16^; and the data for K and L are from^33^.

Study	Species	Country of origin	Sex	Preservation method	Number of mosquitoes in each age group					Total
					≤ 5 days	5 < age ≤ 10	10 < age ≤ 15	15 < age ≤ 20	20 + days	
A	*A*. *arabiensis*	Tanzania	female	fresh	196	302	100	101	172	871
B	*A*. *arabiensis*	Tanzania	female	Silica gel	103	168	103	51	0	425
C	*A*. *arabiensis*	Tanzania	female	fresh	99	50	95	69	0	313
D	*A*. *arabiensis*	Tanzania	male	fresh	100	50	72	67	0	289
E	*A*. *gambiae s*.*s*.	Tanzania	female	fresh	208	305	0	102	93	708
F	*A*. *gambiae s*.*s*.	Tanzania	female	fresh	100	50	93	90	0	333
G	*A*. *gambiae s*.*s*.	Tanzania	female	fresh	100	50	86	94	0	330
H	*A*. *gambiae s*.*s*.	Tanzania	male	fresh	100	50	67	77	0	294
I	*A*. *gambiae s*.*s*.	Tanzania	male	fresh	100	50	76	13	0	239
J	*A*. *gambiae s*.*l*.	Tanzania	female	RNAlater	91	162	42	0	0	295
K	*Ae*. *aegypti*	Australia	male	RNAlater	46	43	46	50	42	227
L	*Ae*. *aegypti*	Australia	female	RNAlater	41	45	46	43	50	225
Total	all				1284	1325	826	757	357	4549

## Results

**Figure 2:**
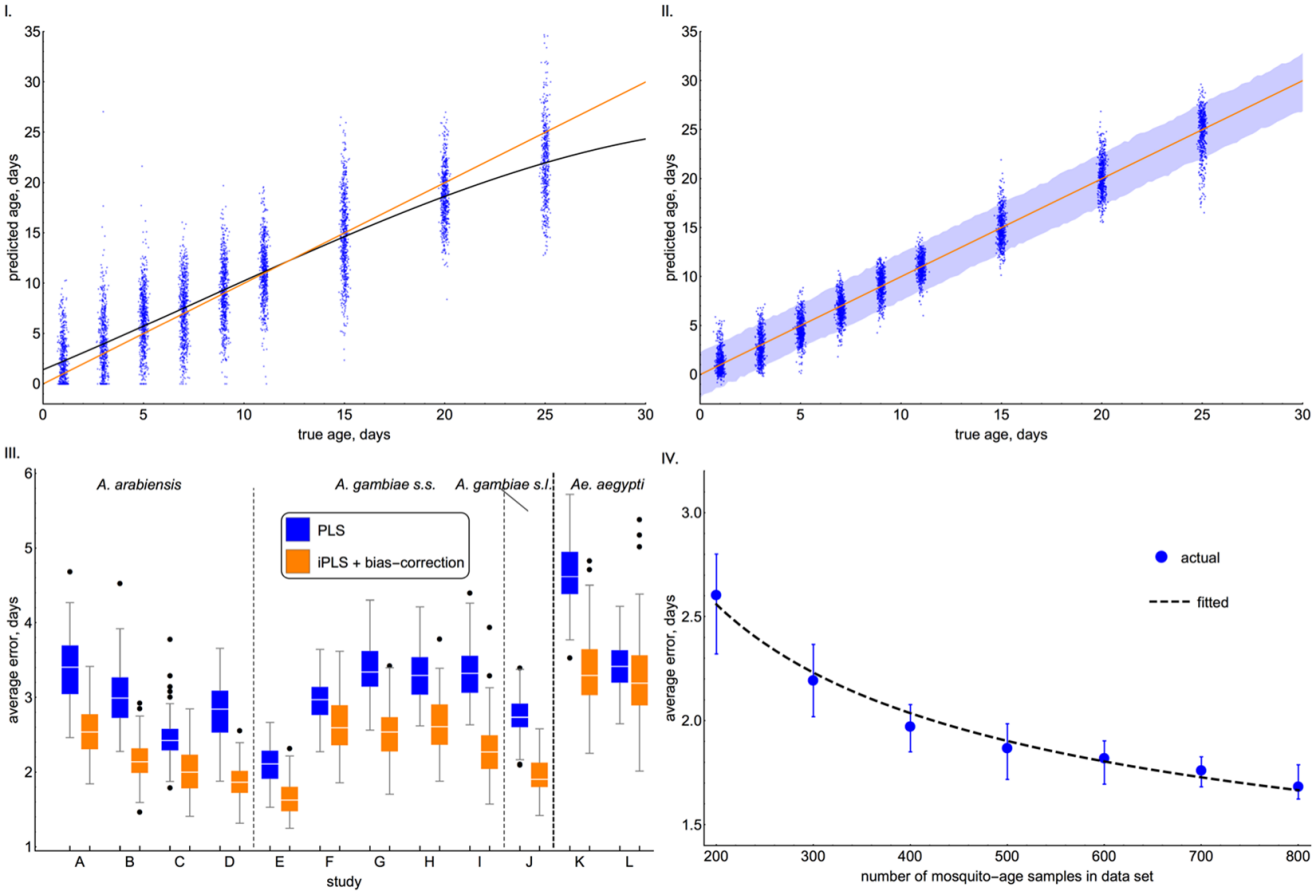
The ability of NIRS to predict the age of individual laboratory reared mosquitoes using standard (**I**) and iPLS (**II** to **IV**) NIRS chemometric methods. I. The accuracy of NIRS in predicting the age of individual Anopheline mosquitoes, using previously published methods to convert spectra into estimates of age. II. Improvements in individual mosquito predictive accuracy and bias reduction through adoption of the iPLS NIRS chemometric methods. In Panels I. and II. the training and testing sets comprised mosquitoes only from study A (see Table 1), the solid black line indicates the fit of a polynomial regression to the data, the blue points indicate the individual-age estimates (with jitter added to the x-axis), whilst orange line shows the ideal (y = x perfect correlation) line. In Panel II., blue shading represents the 95% posterior quantiles for the surrogate model representing the combined action of NIRS and the iPLS machine learning algorithm, and individual age estimates (blue points). Versions of Panel II. are given for each dataset separately (Fig. S1). Panel III. shows the predictions for the individual studies using standard (blue) and iPLS (orange) machine learning methods. In (**III**) the error is estimated by randomly sampling 200 mosquitoes from the training dataset with the middle, lower and upper edges of the boxes representing the 50%, 25% and 75% quantiles in average error obtained across 100 replicates. The lower and upper fences show the 25%/75% quantiles +/− 1.5 times the interquartile range. The dots indicate any outliers, defined as points which lie outside the bounds of the fences. The “average error” is calculated as the root-mean-square error (RMSE) across all replicates. (**IV**) Increasing the number of mosquitoes used in the training dataset substantially increases the predictive accuracy of the age of individual mosquitoes for the iPLS NIRS chemometric method. The solid points represent median average error seen in Study A, and the upper and lower fences represent the 75% and 25% quantiles, each of which was calculated across 100 replicates at each sample size. The regression line here was estimated as: *y* = 0.44 + 34.81 *x*^0.5^, where y is the RMSE and *x* is the number of mosquitoes in the training dataset.

NIRS chemometric procedures that use standard machine learning algorithms to convert spectral data into mosquito age estimates are biased, and typically under-estimate the age of older mosquitoes and overestimate the age in young mosquitoes (Fig. 2I). By changing the machine learning methods to minimise bias and accuracy instead of accuracy alone produces unbiased estimates of mosquito age. Prioritising bias minimisation does marginally increase the overall measurement error. For example, across the whole dataset the average error (Root Mean Standard Error, RMSE, a measure of the average distance between model predicted age and the true value) using the iPLS machine learning method, but maximising accuracy alone, is 2.0 days, whilst minimising bias makes estimates slightly more variable, with a RMSE of 2.2 days. This reduction in the accuracy, however, can be more than accounted for by improving the type of machine learning algorithms employed, by selecting only those parts of the spectra that are known to be predictive (see methods section). Across the whole dataset, using these new iPLS NIRS chemometric methods reduces the error from 3.0 days to 2.2 days on average (Fig. 2II). The combined impact on the average accuracy of the individual studies of minimising bias and improving machine learning is shown in Fig. 2III.

The accuracy of using NIRS to predict the age of individual mosquitoes can be improved by increasing the number of mosquitoes used to train the machine learning algorithm. The accuracy of using NIRS along with the iPLS machine learning methods is estimated to increase along with the square root of the sample size (Fig. 2IV). In our case, this means that for sample sizes greater than roughly 800, there are considerable diminishing returns to including more mosquitoes in the training sample.

The spectra generated from the different studies analysed here are relatively distinct. This is illustrated in Fig. 1II, which shows spectra from mosquitoes with the same age across different studies. If the machine learning algorithm is trained on all but one of the studies, it is currently unable to predict the age of mosquitoes in the excluded study with any accuracy. Similar results are seen when both the training and testing sets comprise mosquitoes from the same species. There is insufficient data to determine whether the inter-study variability is due to using different mosquito populations, the method of mosquito preservation, or some other unmeasured difference between studies. Equally, the machine learning algorithms used here cannot accurately determine the species of mosquitoes from studies not used in the training dataset. There is no evidence of a difference in precision for *Anopheles gambiae sensu strictu* and *Anopheles arabiensis*, though studies directly comparing the two-sibling species in the same location are needed to verify this result.

**Figure 3:**
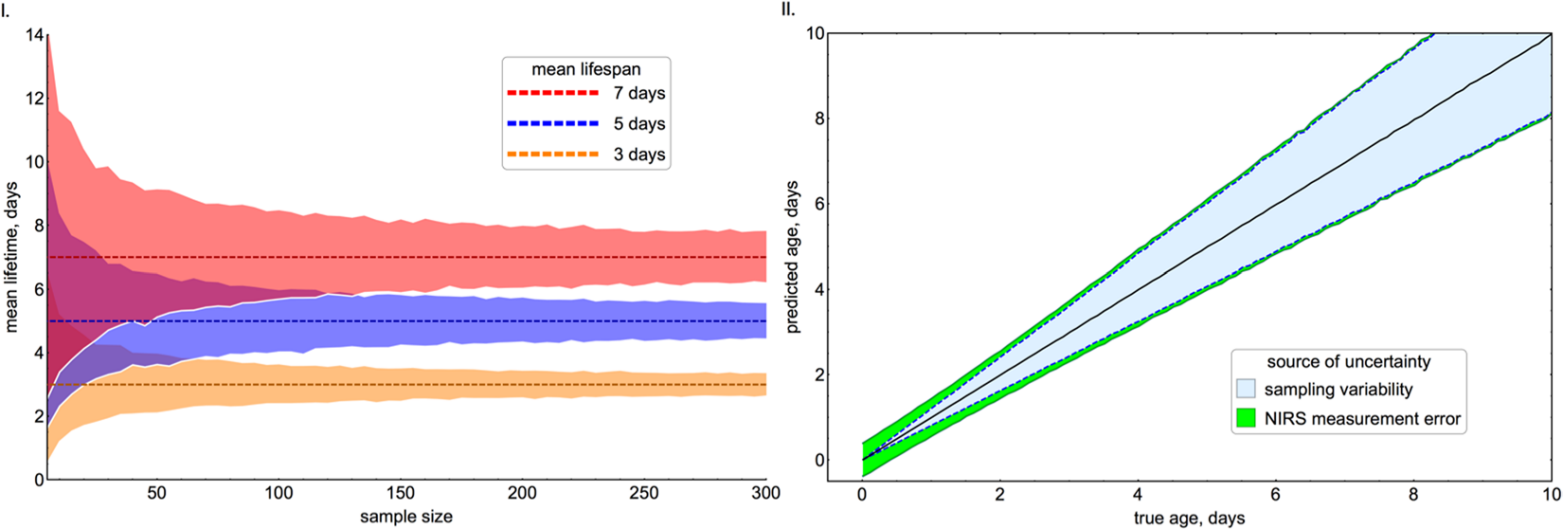
The ability of NIRS to predict the average age of a mosquito population. (**I**) The accuracy of mean population estimates for three *An*. *gambiae s*.*l*. populations of unknown ages when the true average life-expectancy is either 3 days (orange), 5 days (dark blue) or 7 days (red) (approximately 430 mosquitoes used in the training dataset). Dashed lines indicate the true value, whilst shaded area shows the 95% credible interval estimates (95% CI) as the number of mosquitoes sampled from the ‘unknown’ population increases. The data was generated by assuming NIRS errors are the same as those obtained in Study A (see methods). (**II**) The contribution of sampling variability and NIRS accuracy to the overall uncertainty. Shaded area indicates 95% CI caused by sampling variability (dashed line, light blue area) and the additional uncertainty caused by NIRS measurement error (dotted line, green area). Estimates were generated by randomly scanning 100 mosquitoes, with 300 mosquitoes used in the training set.

We now discuss the implications of applying our iPLS machine learning method to training and testing sets composed of mosquitoes from the same study. Whilst NIRS’s ability to predict the age of individual mosquitoes might be relatively poor, if bias is minimised using the iPLS machine learning methods, then the average age of the mosquito population can be estimated with relatively high precision. Increasing the number of mosquitoes with unknown ages sampled substantially increases the accuracy of mean population-level estimates (Fig. 3I). Furthermore, our results indicate that if a reasonable number of mosquitoes are sampled, then small increases in the measurement error of individual mosquitoes (if the estimate remains unbiased), does not substantially diminish the accuracy of population-level predictions (Fig. 3II). This is because most of the uncertainty is generated by sampling error, the random chance a mosquito of a certain age will be caught and scanned. The exact improvement seen by reducing NIRS measurement error will depend on the number of mosquitoes in the training dataset, the average age of the unknown mosquito population, and the number of mosquitoes sampled from it. For example, if the training dataset uses 500 mosquitoes, the unknown mosquito population was on average 5 days old, and 100 mosquitoes were scanned, then NIRS measurement error (as assessed in these studies) would only account for 9% of the overall variability. Having a perfect method of aging mosquitoes would, hence, only increase precision by 9%. Similar results are seen when fewer mosquitoes are sampled (Fig. S2I). The older the average age of the population, the greater the contribution of sampling variability to the overall uncertainty, because the variability in the ages of individual mosquitoes sampled increases (Fig. 3II). NIRS has previously been assessed at a population level by determining the percentage of mosquitoes >7 days old. This metric is less precise than the average age of the population, and does not change linearly with mean-mosquito age (Fig. S2II). More worryingly, the level of bias depends on the measurement error of the aging method making this metric less suitable as a tool for monitoring mosquito populations.

**Figure 4:**
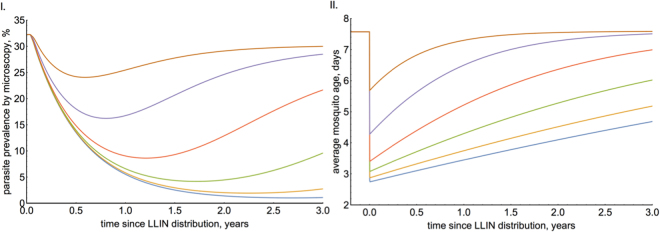
Illustration of how mosquito age could be used to investigate the impact of pyrethroid resistance. A transmission dynamics mathematical model is used to predict how (**I**) human slide prevalence of malaria and (**II**) the mean age of an *An*. *gambiae s*.*s*. mosquito population changes following mass bednet distribution (at 90% coverage) in areas with different levels of pyrethroid resistance. Pyrethroid resistance is measured using a discriminating dose bioassay; be it no resistance (100% mosquito mortality, blue line), 80% mortality (orange line), 60% mortality (green line), 40% mortality (red line), 20% mortality (purple line) or 0% mortality (brown line). See refs^6,23^ for a description of the mathematical model.

Figure 3I indicates that NIRS should be able to differentiate between populations of mosquitoes with an average age difference of two days if > 150 mosquitoes are randomly sampled from each group. To determine whether this difference in mosquito age is sufficient to answer real-world questions, and to further illustrate the utility of the method, a theoretical case-study is provided. Pyrethroids are the only class of insecticides currently used on bednets for malaria control, millions of which are distributed each year. There is growing evidence that mosquito susceptibility to pyrethroids has declined rapidly in some areas of Africa, and risks putting global malaria control in jeopardy^20^. Nevertheless, there is still considerable uncertainty of the current public health impact of pyrethroid resistance, as measuring it directly in the field is technically challenging^21^. Laboratory experiments indicate that resistance might be less of a problem, as mosquitoes which survive pyrethroid exposure in a bioassay or experimental hut trial may die at an elevated rate over subsequent days^22^ reducing their chance of transmitting the disease. To investigate whether pyrethroid treated bednets are still killing mosquitoes in the field, the age distribution of the local mosquito population could be compared using NIRS between two sites, one where resistance is thought to be present and another where resistance hasn’t been detected, following a mass bednet distribution. A transmission dynamics mathematical model of malaria^6,23^ is used to illustrate how the parasite prevalence (Fig. 4I.) and average life-expectancy of a mosquito population (Fig. 4II) may change following mass bednet distribution in areas with different levels of pyrethroid resistance (as assessed using a simple discriminating dose bioassay). Results indicate that the average age of a fully susceptible mosquito population is predicted to drop from >7 days to <3 days immediately following mass bednet distribution. If NIRS age estimates in the field have the same measurement error in as in the laboratory then Fig. 3I. indicates that sampling as few as 50 mosquitoes would be sufficient to differentiate between a susceptible (100% bioassay mortality) and a resistant (20% bioassay mortality) mosquito populations 6 months after bednet distribution. Importantly, changes in the average age of the mosquito population are immediate following bednet distribution, whilst human parasite prevalence takes a number of months before differences between populations can be detected.

## Discussion

NIRS can generate highly precise estimates of the average age of the mosquito population despite its relative inaccuracy for individual mosquitoes. This makes NIRS a promising tool for directly monitoring the efficacy of vector control intervention targeting adult mosquitoes. The ability of an insect vector to transmit disease (the vectorial capacity) is highly sensitive to the death rate of the mosquito^24^. This makes average mosquito age one of the most important measure for assessing community-level protection and the likelihood of a disease outbreak.

As epidemiologists are primarily concerned with population estimates instead of the age of individual mosquitoes, the machine learning methods used to convert spectral data into age estimates can be modified to prioritise minimising bias in individual mosquitoes over just accuracy. Mean estimates can then be improved by increasing the number of mosquitoes sampled. The trade-off between precision and bias will therefore depend on the ease with which mosquitoes can be caught in the wild. This work indicates that the number of mosquitoes required to generate useful estimates are well within the range that can be feasibly collected by routine vector monitoring programmes.

Enthusiasm for the use of NIRS (and other aging methods) has been tempered by the relatively high measurement error seen in published studies. All previous work has used laboratory reared mosquitoes (either colony mosquitoes or from wild caught larvae) which are likely to be more uniform (and give more consistent spectra) than those caught in the wild due to differences in diet, environment and diversity of hazards. It is therefore assumed that NIRS precision will further diminish once these real-life factors are taken into consideration. This work therefore suggests that NIRS is well suited to monitor wild mosquito populations, since population estimates are relatively insensitive to inflations in individual mosquito predictive error, so long as the estimates remain unbiased. More accurate methods of aging individual mosquitoes (with lower measurement error) will make relative little difference to the precision of population-level estimates. This is especially the case in mosquito populations with an average life-expectancy of 3–7 days - the range of age distributions mathematical models predict are most informative for the control of malaria (Fig. 4II) - and likely for other mosquito-borne diseases also.

It is crucial to evaluate NIRS in more natural field settings to assess its use as a tool for routinely monitoring vector populations. This notwithstanding, it is reassuring that simulations which artificially increase the individual error of mosquito age estimates did not substantially reduce overall mean population precision. The average age of wild caught mosquitoes will also be sensitive to biases in trapping techniques, as some methods are thought to preferentially catch certain aged mosquitoes^25^. These biases need to be quantified and adjusted for in any mean estimate of population age. This work also assumed that mosquitoes die at a constant rate in the wild, and the models would need to be adjusted to account for senescence.

Additional studies using NIRS should carefully consider the structure of the training dataset to further improve predictive accuracy. Increasing the number of mosquitoes improves precision, though care should be taken to ensure a broad range of ages, each with the same number of mosquitoes. In the studies analysed here each had fewer older mosquitoes. It is therefore not surprising that the naïve model better fitted young mosquitoes, and underestimates the age of older vectors. Given the epidemiological importance of older mosquitoes a more balanced calibration dataset would be advisable.

Whilst we tried applying a range of machine learning methods including neural networks^26^, random forests^27^, boosted regression trees^28^ and principal components regression to predict the age of samples, we found that iPLS had the best predictive accuracy. The field of machine learning is expansive, however, meaning that it is possible that alternative methods may be better suited to analysing these data than the relatively simple techniques employed here. For example, it is possible that using deep learning methods^29^, support vector machines^30^ or functional regression^31^ approaches could improve the predictive accuracy further. Similarly, other approaches to choose an optimal set of spectral variables exist, and the use of techniques such as the Successive Projections Algorithm, simulated annealing or genetic algorithms have been shown to be useful in other chemometric applications^32,33^. Indeed, we hope that by publishing the dataset of all samples used in this study (available in Supplementary Materials; described in Table 1), that this will encourage others to work to further improve predictive power.

This study is the first time data from different laboratory reared mosquito populations are systematically analysed at the same time. Unfortunately, it is clear from the combined *Anopheles* dataset that for this selection of studies, a calibration dataset generated with one population of mosquitoes cannot be used to predict the age of a different mosquito population. This is not surprising given that the combined dataset was highly diverse, and comprised of studies using different generations of spectrometer with varying fibre optic probe types and light sources. The mosquitoes were also from different mosquito sub-species, locations and times and were killed and preserved using a variety of different methods (for example, fresh, or RNAlater preserved). Further work that standardises the experimental procedures, or refines the machine learning methods, may enable this intra-study variability to be reduced, possibly allowing a universal calibration dataset. If not, then every study may require its own group of mosquitoes with known age in order to train the machine learning algorithm. These calibration datasets could be generated in the field using the F1 generation of wild caught mosquitoes which would encompass the genetic heterogeneity seen in the wild. Nevertheless, the increased investment required to do this would diminish the utility of NIRS for routine vector monitoring, though it would still be useful as a research tool.

NIRS has already proved its ability to differentiate between morphologically indistinguishable *Anopheles gambiae s*.*s*. from *An*. *arabiensis* in wild caught mosquitoes from Tanzania^15^ with relatively good accuracy. Further work is needed to test vector species in other locations, and to determine whether its predictive accuracy can be further improved by refining the machine learning methods currently used for species prediction. PCR is expensive and require specialized training, restricting their use for routine monitoring. If so then NIRS would be ideally placed to be a single method of estimating mosquito age, species and infection status in mosquito populations, replacing the three previous separate dissection and molecular methods which are all laborious and expensive.

## Methods

The paired age-spectra data we analyse comes from five studies of NIRS experiments on mosquitoes (see Table 1 caption), which used a range of different experimental protocols. Prior to scanning, mosquitoes were either anaesthetised and scanned fresh, or killed and preserved (see Table 1 for the preservation method used in each case). In studies A, C, D, E, F, G, H, and I, a QualitySpec Pro spectrometer (350–2500 nm; ASD Inc, Boulder, CO) was used to scan the mosquitoes to generate the spectra; in studies, B, J, K and L were generated using a LabSpec 5000 NIR spectrometer (ASD Inc, Boulder, CO). In all experiments, the mosquitoes were scanned using the method described in^12^, which we briefly summarise: the individual mosquitoes were laid on their backs 2 mm underneath a 3 mm-diameter bifurcated fiber-optic probe, containing 4 collection fibers and 33 illumination fibers. The viewing area spot size was set to approximately 3 mm and was focused on the thorax and head. Each individual spectrum that we used in our analysis was the result of the average of 20 repeat spectra collected by the instrument. We note that, whilst previous studies use the term ‘near-infrared’ to describe the region of the spectrum used in mosquito studies^12–18,34^, the wavelengths considered also include parts of the spectrum in the visible light range.

Previous studies have used GRAMS IQ software to predict individual mosquito age from their corresponding spectra^12–16^, which implements a machine learning method known as ‘partial least squares’ (PLS)^35^. Here, we apply a more recently-developed (albeit related) method to predict mosquito ages from spectra: ‘interval partial least squares’, which has become popular in the field of chemometrics^36^, where it is routinely used to predict chemical composition from spectra. This method involves binning spectra into non-overlapping intervals of specific wavelength ranges, then training separate PLS models that relate data from each interval’s spectrum to an outcome variable. The predictive power from each of the models is then combined using an additive model to produce a final prediction of the outcome.

Here we used the ‘The Graphical iPLS’ toolbox^36^ for Matlab (MathWorks, Inc., Natick, MA) to identify the set of wavelength intervals that was most predictive for mosquito age. This resulted in the following optimal intervals for each genus:

- *Anopheles*: [350–708 nm], [709–1066 nm], [1067–1424 nm],
- *Aedes*: [709–1066 nm],[1067–1424 nm].

The number of PLS components used in the models trained on data from the above wavelength segments was selected by cross validation, using 25 repetitions of bootstrapped sampling. Whilst the optimal number of PLS components per dataset and wavelength segment varied, to limit computational run time and reduce risk of overfitting, we limited the number of PLS components for these models to a maximum of 20.

We combined the models trained on the individual wavelength segments, with a PLS model trained on a wider spectrum [350 nm,1850nm], since the inclusion of the latter was found to improve predictive performance for older mosquitoes. This model was allowed a maximum of 40 PLS components, as higher numbers of components for the wider spectrum models was found to improve predictive performance. The predictions from the separate models were then combined using a generalised additive model with splines (using the ‘gam’ package^37^ in R) to result in a final estimate.

We gauged the predictive performance of a model by training it on one dataset (the “training” set) and using this to predict individual mosquito ages on an independent dataset (the “test” set). The allocation of individual samples into either training or test sets was done in one of two ways: first, we generated training and testing sets by randomly selecting samples produced from within a single experiment; second, we trained a model on data from one experiment and used it to predict mosquito age in a test set composed of an independent experiment. The first approach allows us to gauge the within-study predictive capability of NIRS, whereas the latter approach estimates between-study predictive performance.

For the within-study experiments we selected 70% of overall data to form the training set, and the other 30% comprised the test set. To minimise the risk of overfitting, we then split the training set further into a validation set (30%), and a separate, smaller, training set (the other 70%). This final training set was used to train PLS models on each of the spectral windows, with the individual model predictions on the validation set used as independent variables in training the generalised additive model on the same set (and also for bias correction; see below). The test set was then used to get an independent evaluation the performance of the final model. Using the above methodology we found that there was often over-prediction age for younger mosquitoes, and under-prediction for older specimens (Fig. 2I). This bias in prediction led to a bias in estimating population mean age. Since the individual mosquito predictions were systematically biased, we were able to carry out a correction to our estimates so that the resultant predictions were unbiased across all mosquito ages (Fig. 2II). This bias correction was carried out by fitting a general additive model to the true versus predicted ages in the training data, which was then used to correct the model predictions on the test set. The resultant predictor has a marginally worse average error than the original (uncorrected) model, but as a result is unbiased.

To monitor the effect of interventions, it is most informative to calculate any changes to the mean adult lifespan of the local population of mosquitoes. Here, we estimate the statistical power in using NIRS, alongside our aforementioned machine learning method, in its capacity to estimate the mean age of an *in silico* population of mosquitoes, whose characteristics are known. To carry out this process in its entirety, we would need to generate spectra for the individual *in silico* mosquitoes, which could then be used as inputs to our model. To obviate this need, we instead use a statistical model to act as a surrogate for the combined action of NIRS and our machine learning model. So, the surrogate model takes as input a true mosquito age, and outputs an estimated age that is indicative of the result that would be obtained from using our fitted machine learning model to predict the sample’s age from its NIR spectrum. In particular, the surrogate model that we use here is a Bayesian linear regression model that incorporates heteroscedasticity, which is fitted to the predictions generated by applying our machine learning model to the spectral data (Fig. 2II). Here we assume that the population of mosquitoes has a constant rate of adult recruitment, and that mortality acts in an age-independent fashion. The resultant age structure of the population is exponentially distributed with a mean whose maximum likelihood estimator is the sample mean.

In carrying out an *in silico* investigation of the inferential performance of our machine learning algorithm, we assume that we are able to randomly sample mosquitoes rom the underlying exponential distribution of the population. This assumes that the method of collection for wild mosquitoes does not favour mosquitoes of certain ages. The relationship between the size of the training dataset and RMSE is quantified by fitting a regression line to the observed data. Since the errors in predicting individual mosquito ages is dominated by sampling variation, the (Lindberg-Lévy) central limit theorem implies that the predictive error should decrease with the square root of sample size. This is similar to what we find, because a power law of the form,

1 $$y=a+b{x}^{0.5},$$

fits the data well, where *y* is the median RMSE, x is the number of mosquitoes in the training dataset and parameters *a* and *b* are estimated using maximum likelihood. Mosquito populations are simulated by randomly sampling from an exponential distribution with a known life-expectancy. The observed age distribution of the population is then generated by adding random measurement error sampled from a normal distribution with a standard deviation determined by the size of the training dataset (Equation (1)).

The impact of pyrethroid resistance on mosquito life-expectancy is predicted using a widely-used transmission dynamics model of malaria^6^ which has been reparameterised to account for reduced bednet efficacy caused by pyrethroid resistance (as assessed using a standard discriminating dose bioassay^23^). For simplicity, it is assumed that malaria is transmitted by *An*. *gambiae s*.*s*. mosquitoes, which are at a constant density throughout the year (i.e. there is no seasonality in transmission). Standard pyrethroid bednets are distributed to 90% of the people at time zero and malaria is monitored using slide prevalence in the entire population. The ability of the mathematical model to capture the average age of the population has not been directly validated, though it has been shown to adequately capture malaria dynamics before and after the start of interventions^38^.

### Data availability statement

As part of supplementary materials we make the NIR raw data for this study available, for all studies, apart from A. and E. which are available upon request from the authors.

### Author summary

Mosquitoes transmit malaria, dengue and Zika, which collectively inflict suffering on millions of people each year. The most effective interventions to kill adult mosquitoes are insecticide treated bednets and the indoor spraying of insecticides. The impact of these interventions is under threat due to rapidly growing mosquito resistance to insecticides, and we are in urgent need of methods of evaluating new mosquito control tools in the field. Near infrared spectroscopy (NIRS) shines light through specimens and measures the absorption across a range of wavelengths. As mosquitoes get older, their biochemistry changes, and NIRS, with the use of machine learning techniques, can determine the age of individual mosquito specimens. Here, we compile a database of NIRS experiments carried out on a range of mosquito species and use recently-developed machine learning methods, to considerably boost the predictive performance of NIRS. By prioritising bias minimisation over accuracy alone we can generate precise estimates of the average age of the mosquito population, a very good predictor of the efficacy of interventions which kill adult mosquitoes. A computational malaria transmission model is then used to demonstrate that NIRS can be used to measure the impact of insecticide resistance and the evaluation of control interventions.

## Electronic supplementary material

Supplementary information

Supplementary Dataset
