# Supplementary material for: Monitoring the Age of Mosquito Populations Using Near-Infrared Spectroscopy
Source: Sci Rep. 2018 Mar 27;8:5274. doi: 10.1038/s41598-018-22712-z (PMC5869673; doi:10.1038/s41598-018-22712-z)
Supplement: Supplementary file 1 — Supplementary information [file 41598_2018_22712_MOESM1_ESM.pdf]

Full title: Monitoring the Age of Mosquito Populations Using Near-Infrared Spectroscopy

Ben Lambert[1,2,#], Maggy T Sikulu-Lord[3], Vale S Mayagaya[4], Gregor Devine[5], Floyd Dowell[6], Thomas S. Churcher[2]

[1] Department of Zoology, University of Oxford, South Parks Road, Oxford OX1 3PS, UK

[2] MRC Centre for Outbreak Analysis and Modelling, Infectious Disease Epidemiology, Imperial College London, London W2 1PG, UK.

[3] Queensland Alliance of Agriculture and Food Innovation, The University of Queensland, Brisbane, Queensland, Australia

[4] Ifakara Health Institute, Biomedical Unit, Ifakara and Dar es Salaam Branches, Ifakara and Dar es Salaam, Tanzania.

[5] Mosquito Control Laboratory, QIMR Berghofer Medical Research Institute, Brisbane, Queensland, Australia.

[6] USDA, Agricultural Research Service, Center for Grain and Animal Health Research, 1515 College Avenue, Manhattan KS 66502.

Mention of trade names or commercial products in this publication is solely for the purpose of providing specific information and does not imply recommendation or endorsement by the U.S. Department of Agriculture. USDA is an equal opportunity provider and employer.

All correspondence to ben.c.lambert@gmail.com.

Supplementary Information

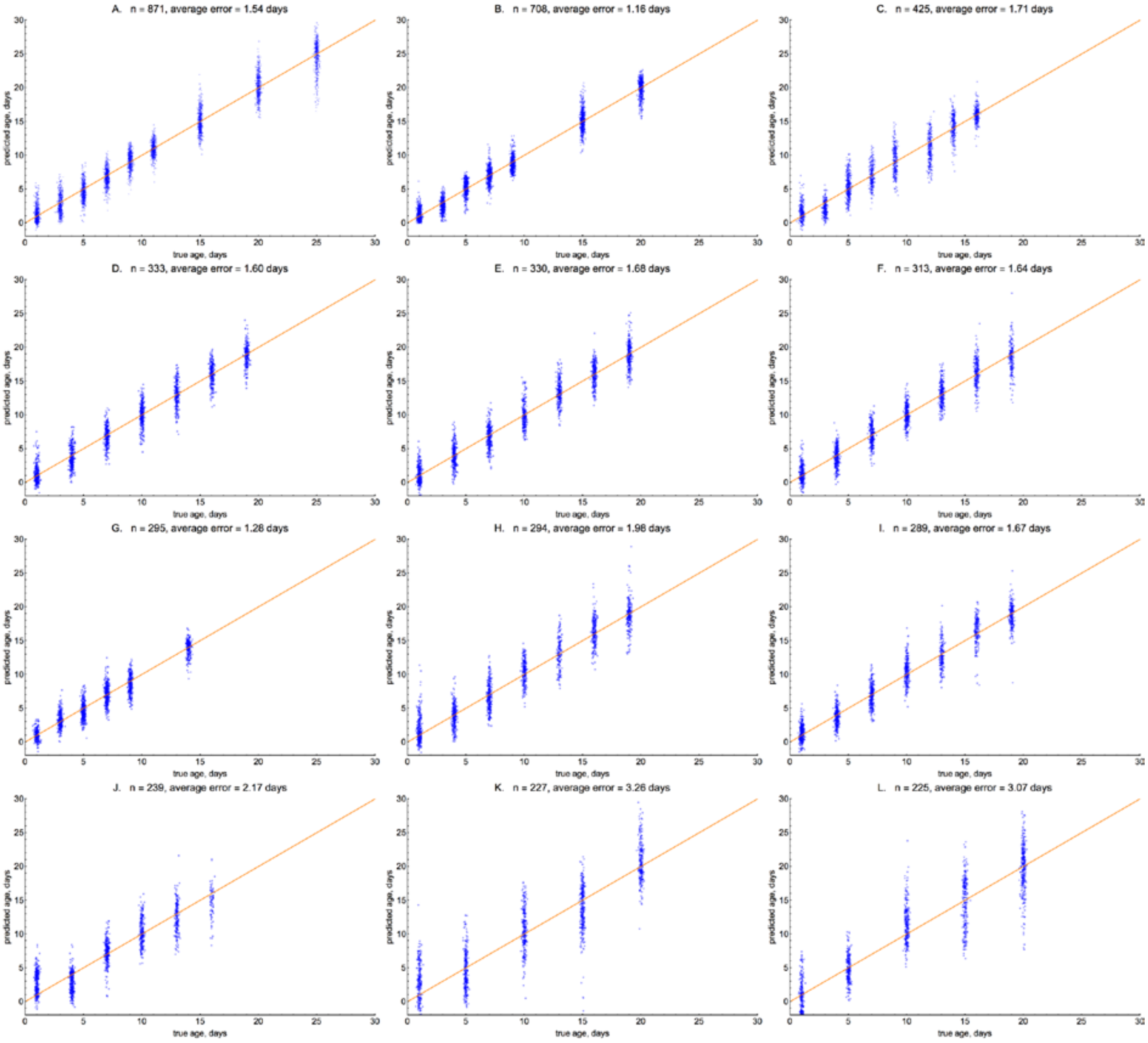

Supporting Fig 1. The ability of NIRS to predict the age of individual laboratory reared *Anopheles gambiae s.l.* (studies A. to J.) and *Aedes agypti* (K. and L.) mosquitoes using novel NIRS chemometric methods.

Figure shows novel predictions of mosquito age on independent test sets for all studies separately, along with the total numbers of individuals in each study. Blue points indicate the individual-age estimates (with jitter added to the x-axis) whilst the orange line which shows the ideal (y = x perfect correlation) line.

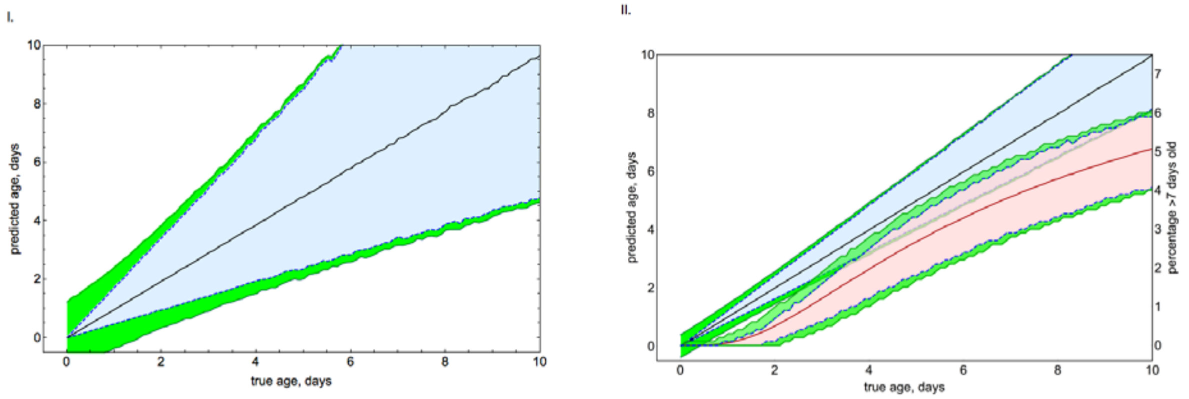

Supporting Fig 2. The ability of NIRS to predict the average age of a mosquito population.
I. The contribution of sampling variability and NIRS accuracy to the overall uncertainty. Shaded area indicates 95% CI caused by sampling variability (dashed line, light blue area) and the additional uncertainty caused by NIRS measurement error (solid green line, outer green area). Estimates were generated sampling (I.) 10 or (II.) 100 mosquitoes (both using 300 mosquitoes in the calibration dataset). In II., the red line shows the relationship between the true mosquito population age and the percentage of mosquitoes >7 days old. Unlike the mean age estimate, the percentage of mosquitoes >7days old estimates are biased by measurement error with red line showing the average estimate once measurement error is considered.
